# Detection and classification of neurons and glial cells in the MADM mouse brain using RetinaNet

**DOI:** 10.1371/journal.pone.0257426

**Published:** 2021-09-24

**Authors:** Yuheng Cai, Xuying Zhang, Shahar Z. Kovalsky, H. Troy Ghashghaei, Alon Greenbaum

**Affiliations:** 1 Joint Department of Biomedical Engineering, North Carolina State University and University of North Carolina at Chapel Hill, Raleigh, North Carolina, United States of America; 2 Comparative Medicine Institute, North Carolina State University, Raleigh, North Carolina, United States of America; 3 Department of Molecular Biomedical Sciences, North Carolina State University, Raleigh, North Carolina, United States of America; 4 Department of Mathematics, University of North Carolina, Chapel Hill, North Carolina, United States of America; 5 Bioinformatics Research Center, North Carolina State University, Raleigh, North Carolina, United States of America; University of Campinas, BRAZIL

## Abstract

The ability to automatically detect and classify populations of cells in tissue sections is paramount in a wide variety of applications ranging from developmental biology to pathology. Although deep learning algorithms are widely applied to microscopy data, they typically focus on segmentation which requires extensive training and labor-intensive annotation. Here, we utilized object detection networks (neural networks) to detect and classify targets in complex microscopy images, while simplifying data annotation. To this end, we used a RetinaNet model to classify genetically labeled neurons and glia in the brains of Mosaic Analysis with Double Markers (MADM) mice. Our initial RetinaNet-based model achieved an average precision of 0.90 across six classes of cells differentiated by MADM reporter expression and their phenotype (neuron or glia). However, we found that a single RetinaNet model often failed when encountering dense and saturated glial clusters, which show high variability in their shape and fluorophore densities compared to neurons. To overcome this, we introduced a second RetinaNet model dedicated to the detection of glia clusters. Merging the predictions of the two computational models significantly improved the automated cell counting of glial clusters. The proposed cell detection workflow will be instrumental in quantitative analysis of the spatial organization of cellular populations, which is applicable not only to preparations in neuroscience studies, but also to any tissue preparation containing labeled populations of cells.

## Introduction

The functional role of a cell is highly dependent on its gene expression, local environment, and external cues [1,2]. The two latter factors are directly related to the spatial location of the cell (e.g., layers in the neocortex, regions in the hippocampus and more). Therefore, in a tissue section, both the number of labeled cells and their spatial distribution are of paramount importance [3–5]. To build these spatial distribution maps, two technologies have been instrumental: (*i*) Sophisticated and automated microscopes (e.g., slide scanners) that facilitate high-throughput data acquisition of biomedical specimens. (*ii*) Tissue labeling methods that include immunohistochemistry, *in situ* hybridization, transgenic reporter mice and more.

**Fig 1:**
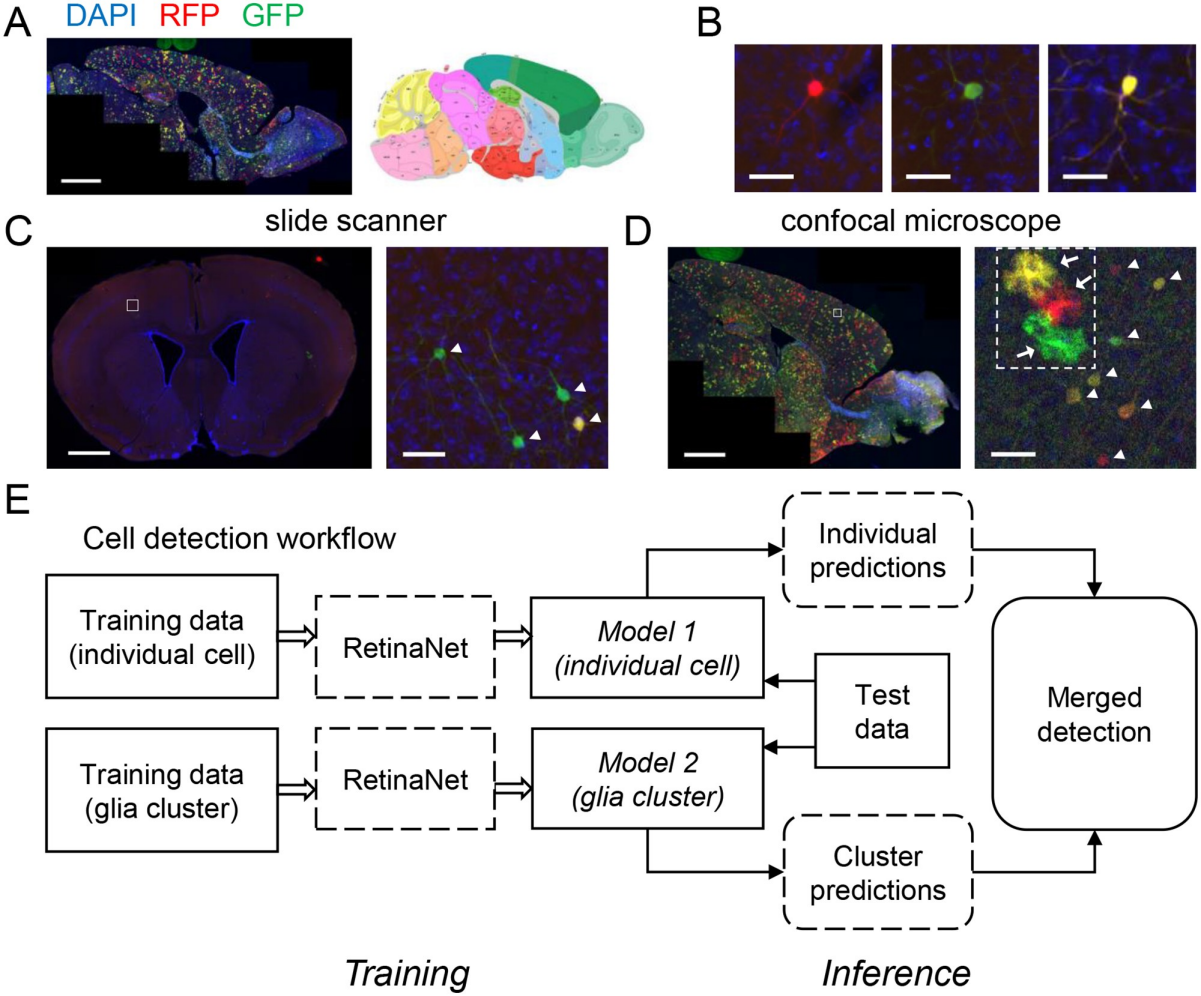
Automated cell and cluster detection in MADM brains. (**A)** Confocal micrograph of a sagittal section from a month-old Nestin-cre, MADM-11 (MADM) mouse forebrain (left) and the corresponding annotated map from the Allen Brain Atlas (right; Image credit: Allen Institute). Scale bar, 1500 μm. GFP–green fluorescent protein, RFP–red fluorescent protein, DAPI– 4’,6-diamidino-2-phenylindole. (**B)** Three isolated neurons captured in the MADM brain where green (enhanced GFP), red (tdTomato) and yellow (both reporters expressed) cells are derived from distinct clones of progenitors earlier during development [8,10,12,13]. Scale bars, 40 μm. (**C)** A representative coronal MADM section with only a single clone of cells labeled and later imaged by a slide scanner (left; scale bar, 1000 μm). Sections were obtained from MADM brains in which red, green, and yellow clones were labeled in the late-stage embryo at very low densities using a Nestin-creER transgene [11]. Boxed area demarcates the zoomed image on the right. Scale bar, 50 μm. (**D)** A representative confocal image of another MADM brain (left; scale bar, 1250 μm) and zoomed image to the right (right; scale bar, 50 μm). Two main types of cells can be seen in C and D: Neurons and glia marked with arrowheads and arrows, respectively. The white dashed frame in D indicates a glia cluster. (**E)** The cell detection workflow. To localize and classify each cell, an object detection network (RetinaNet) was utilized. To detect dense and saturated glia clusters, two RetinaNet models were trained, one to detect individual cells with different colors, and the other to detect only glia clusters. In the inference stage, predictions of individual cells and glial clusters were merged to obtain final output.

Here our goal is to detect and classify cells in images of brain sections obtained from Mosaic Analysis with Double Markers (MADM) mice. MADM allows for simultaneous labeling and genetic manipulation in developmentally derived clones of somatic cells [6]. We and others have extensively used MADM alleles in developmental studies on the roles of various genetic factors, which are involved with neurogenesis [7–9] and gliogenesis [10,11]. An advantage of MADM is that neurons and glia with distinct genotypes are permanently labeled by expression of two fluorescent proteins. Furthermore, MADM labeling occurs in sparse populations such that the entire morphology of individual cells can be easily resolved using microscopy (Fig 1A and 1B). However, in some MADM preparations an entire brain section can contain large numbers of cells despite the sparsity of genetic labeling relative to the total number of cells, which can render manual cell counting tedious and error prone. Hence, the automation of cell detection and classification is vital to boost throughput and unbiased approaches necessary for quantification of complex tissues such as MADM brain sections.

To address this gap, machine-learning algorithms have been utilized to automate and accelerate data processing. Among them, deep learning has outperformed many conventional machine-learning algorithms in multiple tasks e.g., natural language processing, computer vision, speech recognition and more [14]. In computer vision, various deep learning architectures have been employed to address different tasks: For example, convolutional neural networks (CNNs) are used for image classification, Region based CNNs (R-CNNs) are used for object detection, and fully convolutional networks (FCN) are used for semantic segmentation [15]. These deep neural networks (DNNs) have also been applied to biomedical data and showed great promise in, for example, classification of breast cancer histopathology images (CNN) [16], segmentation of whole mouse brain vasculature (3D CNN) [17], and detection of blood cells (You Only Look Once network) [18]. Compared to other network architectures, object detection networks have yet to be extensively used for analysis of biological data. This is surprising as the training of these networks is relatively simple, and their ability to localize and classify targets even in real-time is excellent [19,20]. RetinaNet [21], You Only Look Once (YOLO) [22], Single Shot Detector (SSD) [23], Faster R-CNN [24] are the state-of-art object detection models developed in recent years [20]. Without limitation on the inference time, which can be up to 200 milliseconds, RetinaNet shows superior or comparable results on benchmark datasets [25]. For detection of cells in fluorescence microscopy, recent report have shown that both YOLOv2 and RetinaNet perform well [26]. In our implementation, RetinaNet is selected for cell detection given its slightly better performance on multiple benchmark datasets and its higher performance in dense object detection [21].

Here we present an automatic cell detection workflow using RetinaNet models to analyze brain sections that were obtained from MADM mice (Fig 1C–1E). We put forth training considerations that accelerate the training process and improve model performance. To diversify the training data and generalize our results, images were acquired by either a confocal fluorescence microscope (CFM) or a slide scanner, all of which contained different cellular densities (i.e., genetically different MADM mice). Novel data augmentation methods were also used to compensate for the imbalance in MADM cell numbers due to genotypic differences in the training dataset. To resolve issues with detection and classification of high-density clusters of MADM labeled glia, a unique workflow was designed which incorporated two RetinaNet models, resulting in superior performance compared to a single RetinaNet model trained to detect clusters.

## Materials and methods

### Data generation

MADM-11 mice (The Jackson Laboratory, Bar Harbor, USA; #013751, #013749) were crossed to Nestin-cre mice (bred from MADM-11 mice) using the breeding scheme described previously [11] and harvested at the age of one month under the regulations and approval from the Institutional Animal Care and Use Committee at North Carolina State University. Mice were housed in a 12-h light:dark cycle with ad libitum access to food and water. MADM mice were deeply anesthetized by Avertin overdose (2,2,2 tribromoethanol; 7.5 mg/g body weight), perfused intracardially with 4% paraformaldehyde (PFA) in phosphate buffer saline (PBS, 0.1 M, hereafter), and brains were dissected and submerged in 4% PFA in PBS at 4°C overnight. Brains were embedded in 3% low melting point DNA-grade agarose in PBS and serial 50 μm sections were collected using a vibratome (Leica VT1000S, Leica, Buffalo Grove, USA). Floating serial sections were washed with PBS and blocked for 1 h at room temperature in blocking buffer (10% normal donkey or goat serum, 1% Triton X-100, PBS). Sections were incubated with primary anti-GFP (Green Fluorescent Protein, Abcam, Cambridge, MA; ab13970, 1:2000) and Rabbit anti-RFP (Red Fluorescent Protein, Abcam, ab62341, 1:500) antibodies diluted in 0.1% blocking buffer overnight at 4°C, followed by 3 5-min washes with PBS at room temperature the next day. Alexa Fluor goat anti rabbit Cy3 (Thermo Fisher Scientific, Waltham, USA; A10520, 1:1000), Alexa Fluor goat anti-chicken 488 (Thermo Fisher Scientific, A11039, 1:1000) secondary antibodies were diluted in blocking buffer and incubated with the serial sections for 1 h at room temperature, followed by 3 washes with PBS. Sections were counterstained with the DNA marker (4’,6-diamidino-2-phenylindole; DAPI) at 1:2000 during the secondary incubation. Sections were mounted onto glass slides and coverslipped with Faramount aqueous mounting medium (Dako, Agilent Technologies, Santa Clara, USA). Images of the MADM forebrains were acquired using an FV1000 confocal microscope (Olympus, Waltham, USA) or a slide scanner (VS120, Olympus, Waltham, USA).

### Data annotation

The training data and test data were generated separately from 16 different mouse brain samples (52 brain sections), of which 9 mouse brains were imaged by a slide scanner and the rest were imaged by a confocal microscope. For training, we labeled 2009 individual cells (1219 neurons and 790 glia) and 168 glia clusters from 39 brain sections. For testing, we labeled 551 individual cells (346 neurons and 205 glia) and 48 glia clusters from 13 brain sections. Annotations of individual cells were generated using ilastik version 1.3.3 [27], ImageJ [28], and a customized graphical user interface (GUI) written in Python. The ilastik pixel classification workflow was used to distinguish cells from background for preprocessing. The interactive training process in ilastik allows users to monitor the output and adjust the labels until satisfactory results are obtained. A representative brain section image in the slide scanner dataset was used to train the algorithm in ilastik. After training, batch processing was done on all brain section images. The output probability maps from ilastik were imported into Python to extract centroids of each probable cell region. The centroids were then manually adjusted in ImageJ to precisely locate each cell, add centroids for miss detected cells, and remove false positives. The GUI was used to quickly add labels to each cell. Then the coordinates of fixed size bounding boxes were generated according to centroid locations. A Python script was used to export such annotations into formats compatible with the requirement of RetinaNet.

Glia clusters were annotated in LabelImg (https://github.com/tzutalin/labelImg), a labeling tool in Python. A Python script was used to transform the output format into RetinaNet-compatible format.

### Data augmentation

Color swap was realized by swapping the RFP channel and the GFP channel of each image. During image acquisition, the output signal intensities are proportional to the input laser power. Therefore, to simulate the situation of saturation, the intensities in each channel were multiplied by a factor of 1.5, and a ceiling function was used to emulate saturation. The constant factor of 1.5 was empirically selected.

### Training environments

To train the models NCSU Henry2 cluster was used, as well as UNC Longleaf cluster. For testing, a Lenovo ThinkStation P520 Workstation with one Quadro P1000 graphic processing unit (GPU) was used.

### Object detection model

RetinaNet repository cloned from the source (https://github.com/fizyr/keras-retinanet) was modified for this work (https://github.com/yccc12/keras-retinanet). A pre-trained ResNet50 was used as the backbone. Zero-centering was used as a pre-processing step, as two types of microscopes were used to acquire the data, and zero-centering showed better results in comparison with normalization. Classical data augmentation strategies such as geometrical transforms and noise injection were applied. The initial learning rate was 0.0001 and the batch size was four. Using an Adam optimizer, all the models were trained for 50 epochs, where loss plateaus could be reached. To reduce the effect of randomness, which is inherent to the training process, the training process was repeated three times on the same training data and resulted in three independently trained networks. These networks were tested on the same test data, and their average precision (AP) results were averaged for comparison.

### Area-based counting

For each image patch, the pixels were divided into two groups, pixels that were within an object bounding box and pixels that belonged to the background. For each channel, the mean and standard deviation (SD) of the background (BKG) pixels were calculated. The pixels that belonged to detected neurons within clusters were masked out. Then for each detected glia cluster, the standard deviation of pixels was calculated within each cluster. Afterwards, thresholding was used to detect pixels that belonged to cells within the cluster:

$$\text{Threshold}=\frac{1}{2}\cdot \left({\text{mean}}_{BKG}+{\text{mean}}_{\text{cluster}}\right)+\frac{1}{2}\cdot \left(\text{S}D_{BKG}+SD_{cluster}\right)$$ (1)

After thresholding, morphological opening and closing were conducted to extract the area of the clustered glia above the threshold. The extracted area was then used to estimate the number of cells in the region, simply by dividing the area by 2000 μm^2^ and rounding the result.

The root-mean-square error (RMSE) was used to evaluate the area-based counting. For each color of glia, we calculate the RMSE as below.


$$RMSE=\sqrt{\frac{\sum_{i=1}^{M}{\left({\hat{n}}_{i}-n_{i}\right)}^{2}}{M}}$$ (2)

$\hat{\text{n}}$ is the estimate number of glia in the cluster. n is the ground truth number. M is the total number of detected glia clusters.

### Evaluation metrics

In object detection tasks [29], precision is defined as: the number of correct predictions, also named as true positives (TPs), divided by the number of all predictions. Higher precision means fewer false positives (FPs).


$$\text{Precision}=\frac{TP}{TP\;+\;FP}$$ (3)

Recall is defined as the number of correct predictions divided by the number of all ground-truth positives i.e., including false negatives (FNs). Recall illustrates the sensitivity of a model, and high recall value indicates low number of false negatives.


$$\text{Recall}=\frac{TP}{TP\;+\;FN}$$ (4)

To comprehensively measure these two aspects of a model, F-score (F_1_) is utilized [30]. Similar to precision and recall, the F-score values are ranging from 0 to 1. Value of 1 indicates that the predictions and ground-truth annotations are identical.


$$F_{1}=\frac{2\cdot \text{Precision}\cdot \text{Recall}}{\text{Precision}+\text{Recall}}$$ (5)

To evaluate the performance of an object detection model, we followed the average precision measure [31], in terms of the relative overlap of the bounding boxes. During the inference stage, an object detection model will output per prediction the coordinates of a bounding box, a classification label, and a confidence score. For each class, the output predictions are first ranked according to their corresponding confidence scores. Then a set of precision and recall will be calculated following the confidence rank. A prediction is true if the intersection over union (IoU) of the bounding boxes of the prediction and an undetected object is above 0.5, where the IoU is defined as the area of intersection divided by the area of union given two bounding boxes.

Starting from the prediction with the highest confidence score, the first pair of precision and recall are calculated based on this prediction only. Then adding the prediction with the second highest confidence score, the second pair of precision and recall are calculated considering top two predictions, and so on at each rank. Finally, a set of precision and recall values are obtained, and an initial precision-recall curve is depicted in which each point is a pair of precision and recall. For each recall value r, the precision value p_r_ is replaced by the maximum precision value p_r’_ whose corresponding recall value is no less than the original recall value (r’ ≥ r). The AP of the class is obtained by calculating the area under the adjusted precision-recall curve. For multiclass detection, the overall AP is an average of AP values across all classes.

### Statistical analysis

Unpaired t-tests assuming unequal variances were performed in Microsoft Excel.

## Results

### Training configuration experiments

A RetinaNet model was trained to detect six classes of individual cells: green glia, yellow glia, red glia, green neuron, yellow neuron, and red neuron. In addition to the conventional parameters (e.g., optimization hyper parameters) our training approach was adapted to account for the unique properties of our datasets. We designed three different training configurations and tested their effects on the network’s performance: (*i*) Adding a DAPI channel, which reveals stained cell nuclei, as an input to the network. (*ii*) Training the network without background tiles. (*iii*) Adopting a color-independent detection approach to classify cells. In all the comparisons between the models, an average precision (AP) measure was used [31].

**Fig 2:**
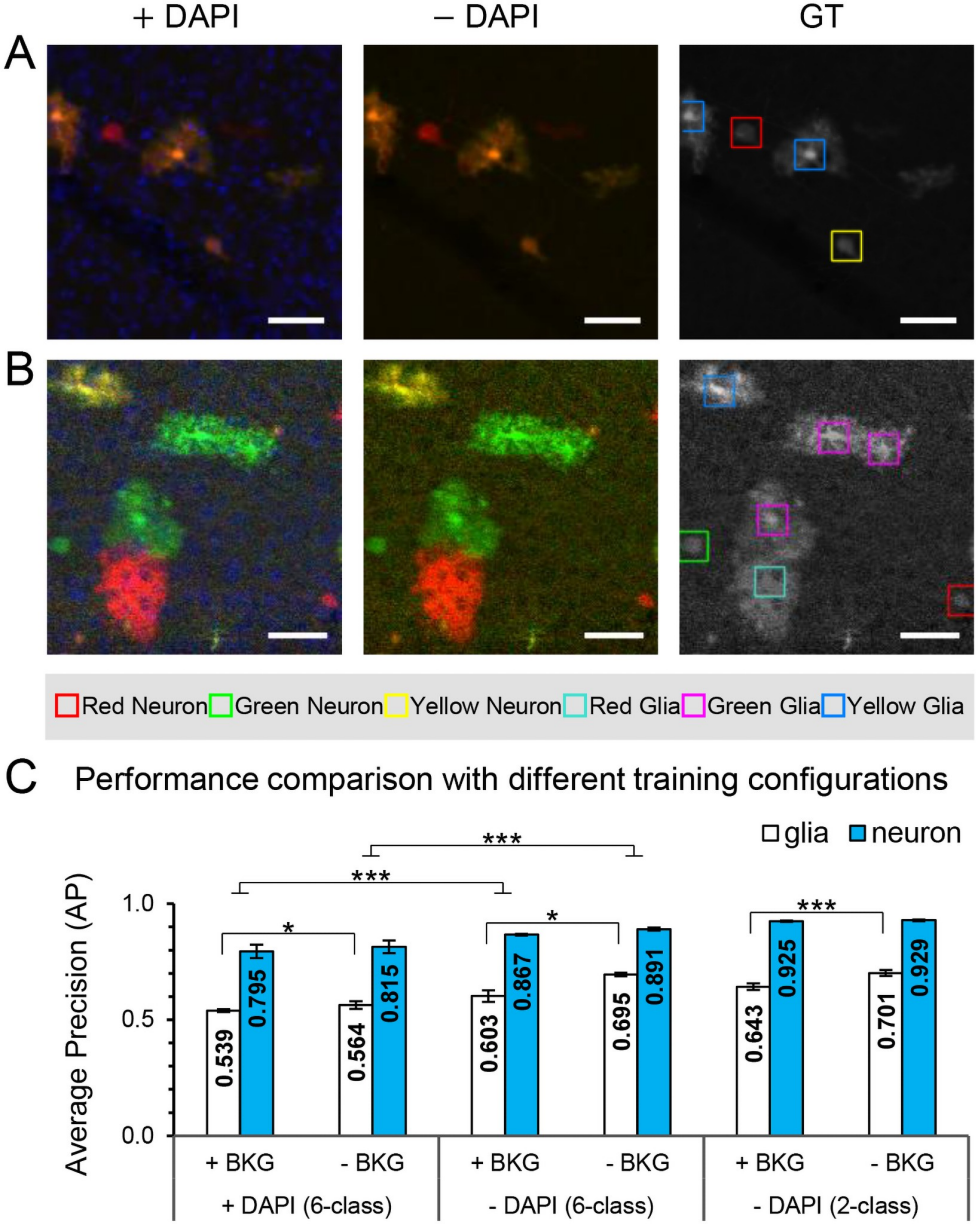
Training configurations that influence the performance of a single RetinaNet model in individual cell detection. (**A, B)** Representative images from the slide scanner and the confocal fluorescence microscope (CFM) respectively, with the corresponding ground truth (GT) annotations. The GT annotations are on grayscale images for clearer display of the bounding boxes. Scale bars, 50 μm. (**C**) Average precision (mean ± SD, n = 3; *, p < 0.05; ***, p < 0.005; unpaired t-test) of trained models with different training configurations. Configurations include training: (*i*) With and without the DAPI channel. (*ii*) With and without pure-background (BKG) image patches (i.e., no target cells in the image patches). (*iii*) On six classes (red/green/yellow neuron, and red/green/yellow glia) versus two color-independent classes (neuron, glia). Training with the DAPI channel and BKG fail to improve the performance and degrade the average precision. Two-class detection shows better performance on neurons without color classification. Please note that when the significance line ends with inverted T, it shows significance between the average of the two classes.

Both the slide scanner (Fig 2A) and confocal (Fig 2B) datasets were acquired on three color channels: GFP (green), RFP (red) and DAPI (blue). While GFP and RFP reporters are genetically expressed in a subset of cells permanently, the DAPI stain binds to DNA inside all the cells in a sample. Therefore, an experiment of evaluating the network’s performance with or without a DAPI channel was conducted. Training without the DAPI channel significantly improved the model performance of six-class detection from 0.667 ± 0.017 to 0.735 ± 0.013 (mean ± SD, p < 0.005, unpaired t-test, n = 3; Fig 2C). It may be possible that the lack of cell-specificity inherent to the DAPI channel obscured successful detection of the red and green channels.

When generating the training data, a full brain section image was cropped into small image patches. As a result, in a sparse sample, there were many pure-background image patches (i.e., patches without any cells), whereas the target cells were distributed sparsely across the tissue. For instance, in a sparse section there were 70 image patches of pure background compared to 33 image patches that contained target cells (Fig 1C). Such pure-background image patches were considered as negatives during training, but failed to improve and even slightly degraded the performance of the model (Fig 2C). Therefore, we decided to train without pure background patches hereafter.

Next, we trained a RetinaNet model to detect only two classes, neurons and glia regardless of their MADM colors. Given the same training data, reducing the number of classes led to better performance, mainly for the neuron class from AP of 0.891 ± 0.007 to 0.929 ± 0.003 (mean ± SD, p < 0.05, unpaired t-test, n = 3; Fig 2C). This improvement in performance was likely to result from the imbalance of different colors in the training data. It should be noted that for comparison with the two class models, we averaged the AP values of three colors for either neurons or glia in the six-class model.

### Data augmentation for improved detection of individual cells

**Fig 3:**
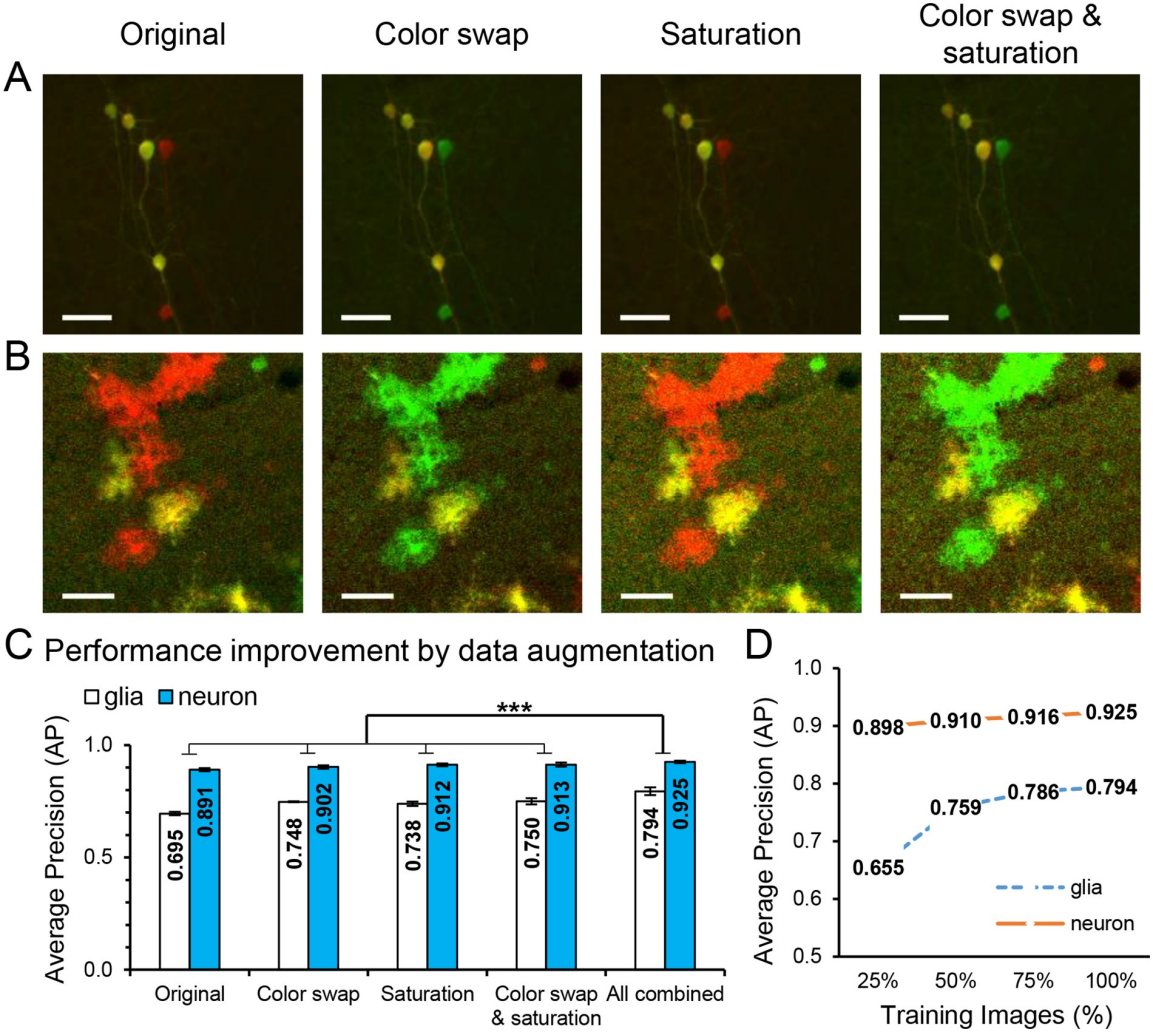
Data augmentation by color swap and saturation improve individual cell detection in a single RetinaNet model. (**A, B)** Slide scanner and confocal images respectively with data augmentation including color swap and/or saturation. Scale bars, 50 μm. (**C**) Average precision results (mean ± SD, n = 3; ***, p < 0.005; unpaired t-test) from the different augmentation conditions. Utilizing each type of data augmentation provided similar results. Harnessing all three augmentations together led to the best performance in both neuron and glia detection. (D) Average precision results with respect to proportion of training images. A plateau of performance was reached when increasing the amount of training data.

To match the performance of the six-class model to the two-class model, we aimed to eliminate color dependent biases in the training data. Toward this end, we doubled the size of the training set by adding a color swapped version of the original (Fig 3). We used the color swap approach since all the neurons independent on their colors looked very similar, and this was also true for glial cells. We also found that the saturation of the input data might be color-dependent, hence we multiplied the original image by a constant and used a ceiling function to emulate saturation (see Methods section). After training with each type of augmentation condition, we found that data augmentation improved the detection of individual cells. Utilizing all types of data augmentation together led to the best performance of individual cell detection with AP of 0.860 ± 0.006 (mean ± SD, p < 0.005, unpaired t-test, n = 3; Fig 3C), which exceeded the performance of the two-class model with AP of 0.815 ± 0.005 (mean ± SD, n = 3) while maintaining the ability to distinguish MADM colors. This result reiterated the importance of tailoring the data augmentation to the unique properties of the dataset. Moreover, numerical experiments were performed to evaluate the model’s performance with different amount of training data (Fig 3D). Fig 3D shows that as the amount of training data increased, a plateau of performance was reached.

### A single RetinaNet model reached an average AP of 0.90 on individual cells

After fine-tuning a RetinaNet model using the data augmentation techniques presented in Fig 3, we tested the model performance on an independent test set i.e., the test set was derived from different mouse brain samples from those used for training. Table 1 summarizes the AP of individual cell detection across six classes and after averaging the test results from three independent networks, which were trained on the same dataset. Detection of neurons reached an AP of 0.943 ± 0.005 (mean ± SD, n = 3), while the glial cells reached an AP of 0.857 ± 0.002 (mean ± SD, n = 3). The precision-recall curves of the best model are shown in S1 Fig. Moreover, the method was evaluated using 5-fold cross validation where an AP of 0.90 was obtained (Table 2). For completeness, we also compared the results of the two-class model (neurons and glia regardless of their MADM colors) after data augmentation with the six-class model. The two-class detection model had an average precision of 0.952 ± 0.005 (mean ± SD, n = 3) for neurons and 0.863 ± 0.011 (mean ± SD, n = 3) for glial cells. These are comparable results to the six-class detection model, but this comparison is doing a disservice to the six-class model, since on top of detecting neurons and glia, it also had to classify the cells by color.

**Table 1:** Average precision results (mean ± SD, n = 3) across six classes in individual cell detection using a single RetinaNet model.

	Neuron	Glia	Average
Yellow	0.950 ± 0.005	0.876 ± 0.011	0.913 ± 0.008
Green	0.955 ± 0.006	0.849 ± 0.017	0.902 ± 0.007
Red	0.925 ± 0.004	0.847 ± 0.001	0.886 ± 0.002
**Average**	**0.943 ± 0.005**	**0.857 ± 0.002**	**0.900 ± 0.001**

**Table 2:** Average precision results (mean ± SD, n = 5) of 5-fold cross validation across six classes in individual cell detection using a single RetinaNet model.

	Neuron	Glia	Average
Yellow	0.932 ± 0.028	0.857 ± 0.023	0.895 ± 0.022
Green	0.953 ± 0.030	0.850 ± 0.112	0.902 ± 0.052
Red	0.947 ± 0.013	0.880 ± 0.012	0.914 ± 0.008
**Average**	**0.944 ± 0.018**	**0.863 ± 0.037**	**0.903 ± 0.016**

**Fig 4:**
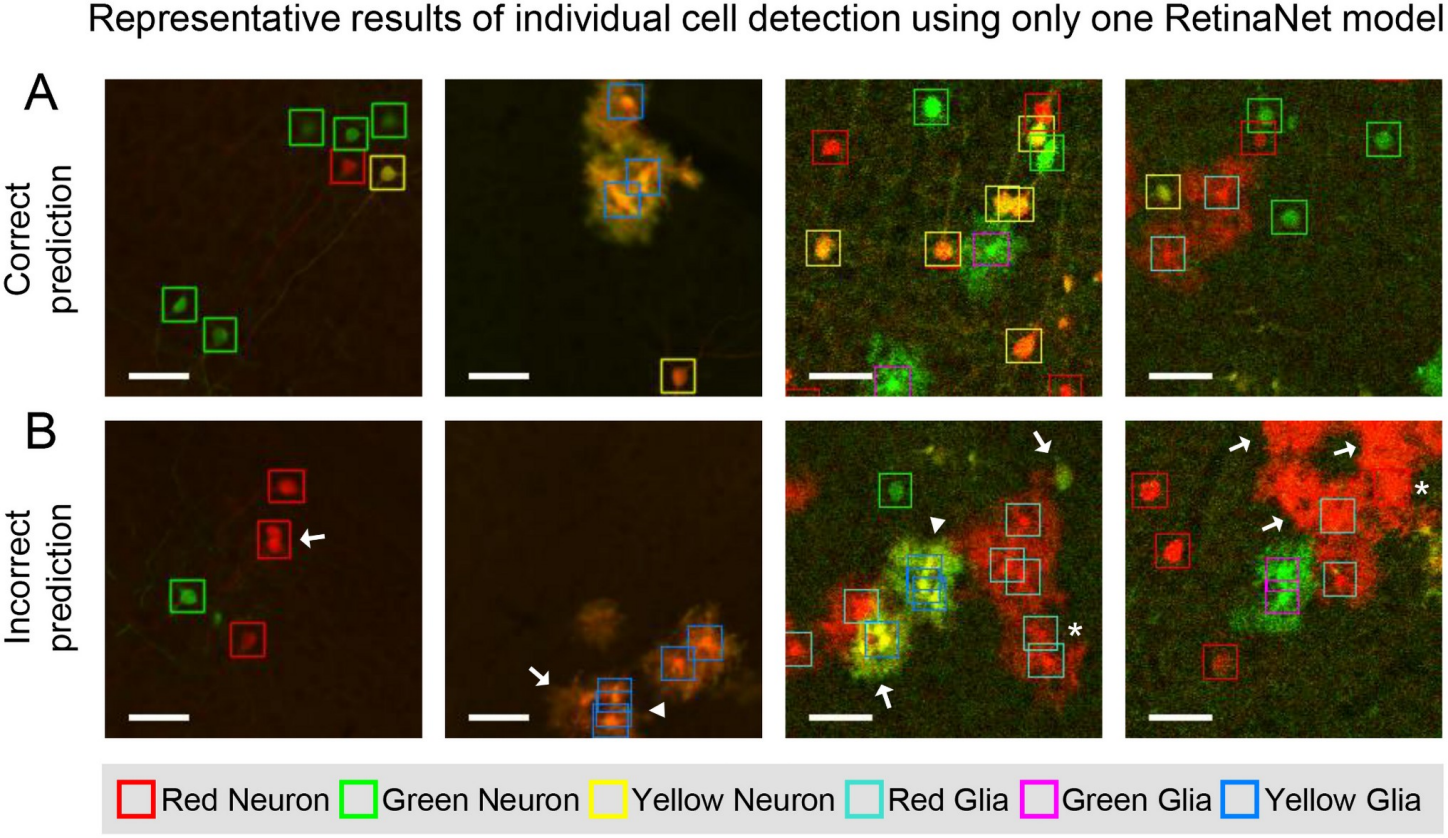
Representative model-detected neurons and glia. (**A)** Examples of correct predictions on images from both the slide scanner (first and second images) and confocal (third and fourth images). (**B)** Examples of incorrect predictions from the trained model. Three main error types including miss detection (arrow), redundant detection (arrowhead) and false detection (asterisk) are marked in the images. Glial clusters and their saturation were the main factors that caused false predictions and miss detections. Threshold of confidence was 0.5 in all images. Scale bars, 50 μm.

To further improve these results, we studied cases in which the model made correct (Fig 4A) and incorrect (Fig 4B) predictions. Based on observations, we reasoned that the larger inherent variability in the glial cells’ morphology, and their tendency to form dense clusters underlay the better performance achieved by RetinaNet in identifying neurons versus glia. This discrepancy is exacerbated when the glial clusters are saturated as a result of higher density of glial processes per pixel. Therefore, we hypothesized that independent detection of glial clusters may improve the performance of the model.

### Merging results of two RetinaNet models improved detection and classification of glia

We found that the detection of individual glial cells in a cluster was difficult for the network, as it is for a manual annotator. To address this issue, we first made an attempt of training a single RetinaNet model to detect seven classes: the previous six classes and an additional glia cluster class. In the training data of seven-class detection model, glia clusters with various sizes were annotated separately from isolated individual glia. Overall, we found that this approach failed to perform well in detection of glial clusters.

Next, two RetinaNet models were trained (Fig 1E), one for individual cells (see Table 1 for AP results), and one to solely detect glia clusters. The AP of glia cluster detection was 0.76 on an independent test set. Lower AP is expected for glia clusters due to variability in the glia clusters in terms of size and morphology, which hinders consistent annotation of bounding boxes even for a manual annotator. Then the results from the two models were integrated using a rule-based merging process. The three rules are: (*i*) Keep all detected clusters with a confidence score above 0.5. (*ii*) Keep glia clusters with confidence above 0.3 that have overlapping individual glial cells and remove these individual cells. In such cases, the individual cells provide evidence that increases our confidence that a cluster is present. (*iii*) Eliminate redundant clusters–i.e., when more than half of the bounding boxes for nearby clusters overlap.

**Fig 5:**
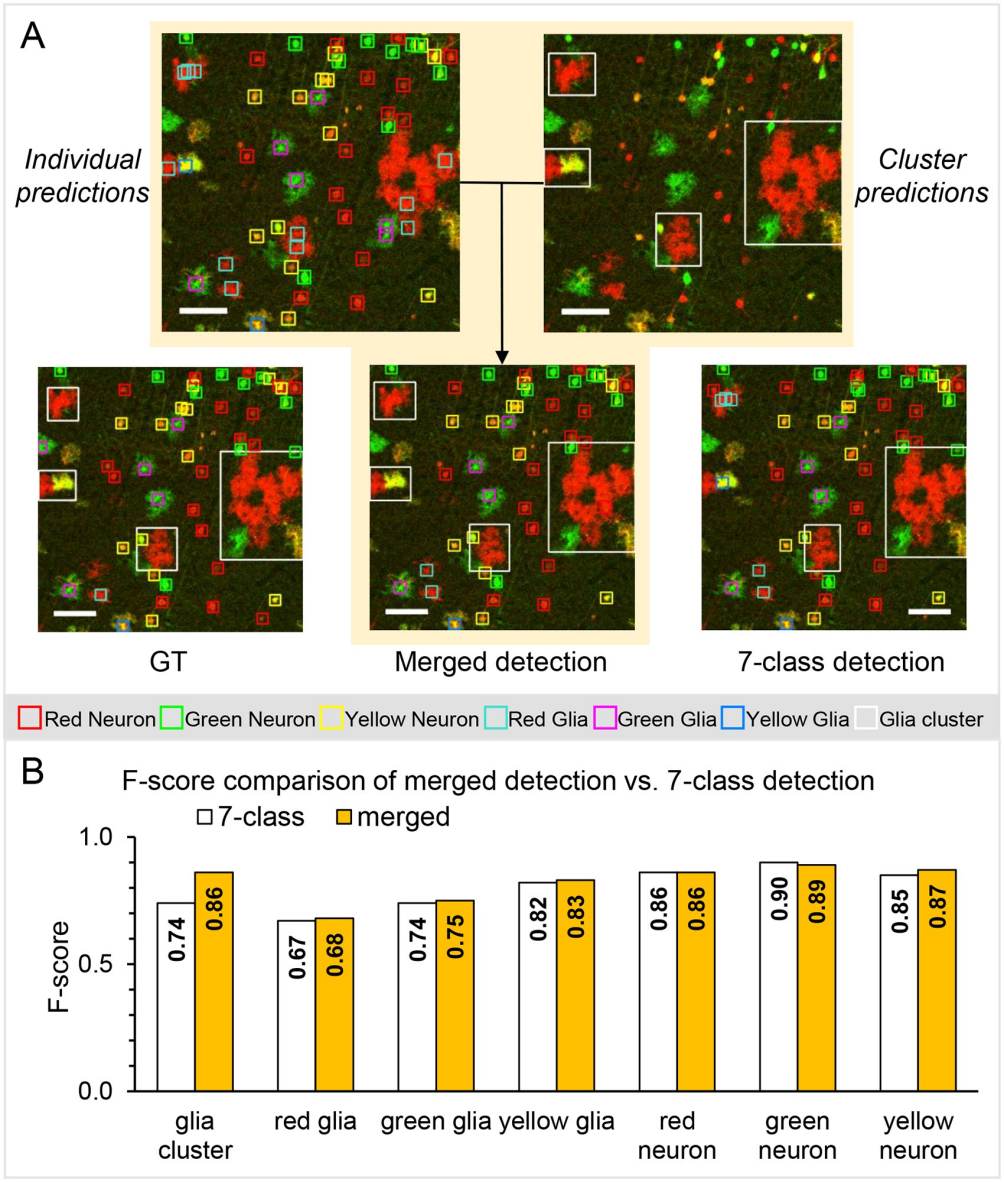
Combining predictions from two RetinaNet models enhances performance. (**A)** An example of merging results compared to the ground truth (GT) annotations. As shown in Fig 1E, two RetinaNet models were trained separately: One for individual cell detection and one for glia cluster detection. Predictions from both the trained individual cell-detection models and the trained glia cluster-detection model were then merged to assess performance. For comparison, an additional RetinaNet model was trained to detect seven classes simultaneously (glia cluster, red/green/yellow glia, and red/green/yellow neuron). Predictions on the same image patch with confidence scores above 0.5 are shown. Scale bars, 100 μm. (**B)** F-score distributions for merged detection and seven-class detection on the test dataset. F-score is a comprehensive measure of accuracy combining precision and recall. F-score in the glia cluster improved significantly by merging predictions from the two RetinaNet models.

Examples of the merging process are shown (Fig 5A and S2 Fig) with the corresponding ground truth and the seven-class detection results. To evaluate the results, we merged ground truth annotations according to the same aforementioned rules and calculated the F-score for each class on the independent test set. The merged results consistently showed higher F-scores in glial classes than using the seven-class detection network, especially on the saturated images (Fig 5B). Particularly, the F-score for detecting glia cluster was higher using the two RetinaNet models versus the seven-class network (0.86 and 0.74, respectively).

To determine the number of glia within a detected glia cluster, we used area-based cell counting to estimate the number of individual cells. Cell regions were extracted by thresholding and morphological operations, and the number of individual cells was then estimated according to the area of the extracted cell masks (see Methods section and S3 Fig). The root-mean-square error (RMSE) was used to evaluate the counting results of the detected clusters. Counting results obtained for 23 test glia clusters, show that the results of merging the two RetinaNet models were better than six-class detection with a RMSE of 0.59 compared to 1.43 in red glia. Please note, that the red glia was the most common type of cluster in this specific dataset. For green and yellow glia, the counting in glia clusters by merging the two RetinaNet models had RMSEs of 0.36 and 0.69 respectively, which was comparable to the six-class detection with RMSEs of 0.21 and 0.36 respectively.

## Discussion

In this study, we developed an automatic cell detection workflow that was applied to images obtained from MADM-labeled mouse brain sections. Our workflow achieved an overall AP of 0.90 ± 0.001 (mean ± SD, n = 3) for individual cell detection, with an AP of 0.943 ± 0.005 and 0.857 ± 0.002 for individual neurons and glial cells, respectively. We also trained YOLOv3 and SSD models instead of RetinaNet to detect individual cells, and the best AP results that we achieved were 0.787 and 0.793, respectively. Please note that we did not spend extensive amount of time optimizing the models as compared to RetinaNet. To detect dense and slightly saturated glia clusters, we incorporated an additional RetinaNet model. This approach showed superior performance in comparison with a more traditional approach, i.e., a single RetinaNet model with seven classes (individual cells plus glia clusters). We also presented a novel data augmentation method that was used to compensate for color-, intensity-, and saturation-dependent biases in the dataset due to the investigated genotype and acquisition conditions. To the best of our knowledge, this is the first paper to integrate multiple fluorescence channels (except the DAPI channel) and to use the RetinaNet model for color classification. We believe that the presented approach could be used in multiple tissue preparations and in quantification of various structures with double stain. This is especially appealing as the training of detection networks is fast and relatively simple.

Although in the current stage of its development our workflow has shown great promise, it still faces several limitations. First, deep learning approaches are black box methods, i.e., it is difficult to explain the relationship between the input and the output, and therefore improve their performance in scenarios where they fail. It will be interesting to explore and utilize explainable artificial intelligence (XAI) methods, which can be interpreted by humans [32–34]. However, XAI still faces many challenges to obtain explainability in deep learning models [32]. Second, the presented approach did not explore the multiscale capability of the RetinaNet model, i.e., detection under different magnification conditions. Please note that magnification in this context is not related to structural hierarchies or different orders of structure [35,36], magnification relates to the ability of a microscope to produce a larger image of the object, relative to its actual size. This multiscale capability provides flexibility to integrate datasets that were acquired with different magnifications. Here, the multiscale capability of the RetinaNet model was not tested, since the acquired datasets had similar magnification. Future work will introduce more variability to our datasets, by including data with different magnifications, and from additional imaging modalities such as light-sheet fluorescence microscopy. Third, detecting individual glial cells using a 10 × magnification (~ 0.3 numerical aperture) in a cluster is challenging, not only for a machine but also to a manual annotator. Therefore, other than counting cells within the cluster using area-based cell counting, we will reimage the dense and challenging regions with higher resolution e.g., 40 × magnification (~ 0.8 numerical aperture), and high-dynamic-range acquisition. Additionally, we plan to integrate an object detection network into a microscope in the near future. This will enable the real time detection of problematic regions, and in turn allow for local reimaging at higher magnifications whenever further resolution of these ambiguous cases in datasets is needed. Such need-based approaches of automatic acquisition will translate into an efficient way to utilize expensive microscopes and to compress the raw dataset sizes.

Last, the double marker approach in our case provided the cell genotype and the markers/colors should spatially overlap. However, in other applications such as diagnosis of non-small cell lung cancer [37] the intracellular localization of the double markers might reveal important information. In these cases, segmentation of the markers will be required, thus revealing a potential limitation for object detection approaches.

## Supporting information

S1 Fig Precision-recall curves across six classes in individual cell detection. (TIF)

S2 Fig Examples of merging results compared to the ground truth (GT) annotations. **(A, B)** Representative merging results of the images acquired from the slide scanner and the confocal fluorescence microscope (CFM), respectively. Two RetinaNet models were trained separately: One to detect individual cells and one to detect glia clusters (Fig 1E). Predictions of individual cells and glia clusters were then merged to evaluate the performance. For comparison, a RetinaNet model was trained to detect seven classes simultaneously (red/green/yellow neuron, red/green/yellow glia, and glia cluster). Predictions on the same image patches with confidence above 0.5 are shown. Note that based on the merging rules, cluster predictions with confidence above 0.3 are also considered in the merging process. Scale bars, 100 μm.
(TIF)

S3 Fig Representative area-based counting within glia clusters. **(A, B)** Counting results of glia clusters from images acquired using a slide scanner and a CFM, respectively. Binary masks of cells regions were generated for each color by thresholding and morphological operations. Estimated cell numbers of each color are marked in the images. The glia cluster in A contains 2 red glia and the glia cluster in B contains 9 red glia, 2 green glia and 1 yellow glial cell according to ground truth annotations. Scale bars, 25 μm.
(TIF)
