# Supplementary figures and images for: Detection and classification of neurons and glial cells in the MADM mouse brain using RetinaNet
Source: PLoS One. 2021 Sep 24;16(9):e0257426. doi: 10.1371/journal.pone.0257426 (PMC8462685; doi:10.1371/journal.pone.0257426)

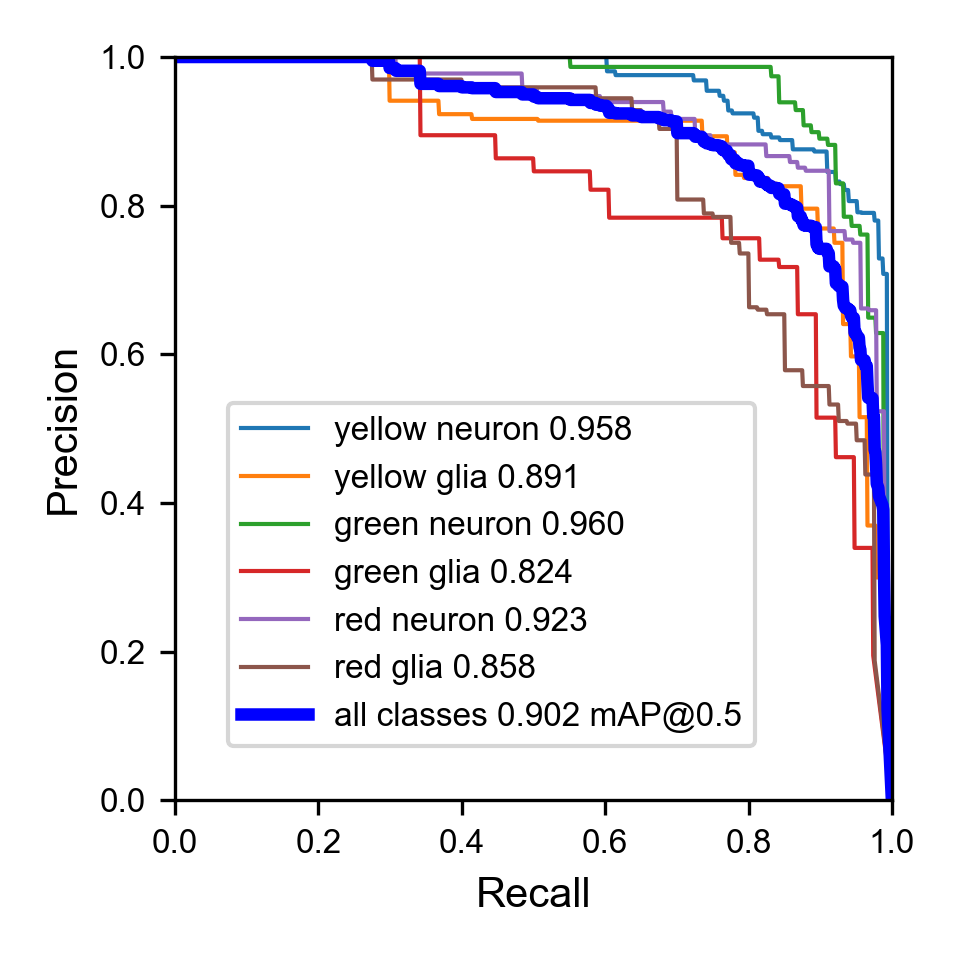

Supplement: S1 Fig — (TIF) [file pone.0257426.s001.tif]

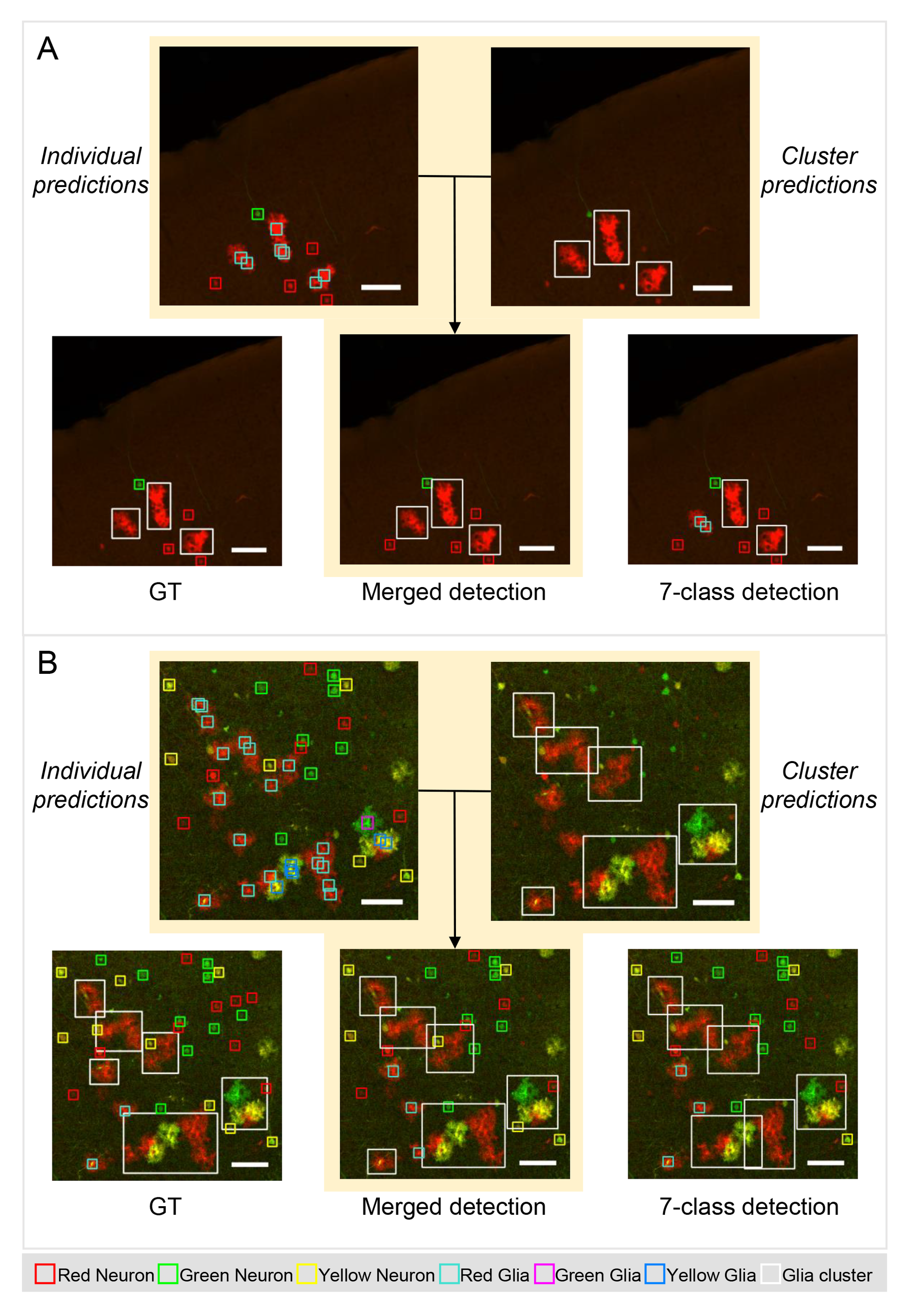

Supplement: S2 Fig — (A, B) Representative merging results of the images acquired from the slide scanner and the confocal fluorescence microscope (CFM), respectively. Two RetinaNet models were trained separately: One to detect individual cells and one to detect glia clusters (Fig 1E). Predictions of individual cells and glia clusters were then merged to evaluate the performance. For comparison, a RetinaNet model was trained to detect seven classes simultaneously (red/green/yellow neuron, red/green/yellow glia, and glia cluster). Predictions on the same image patches with confidence above 0.5 are shown. Note that based on the merging rules, cluster predictions with confidence above 0.3 are also considered in the merging process. Scale bars, 100 μm. (TIF) [file pone.0257426.s002.tif]

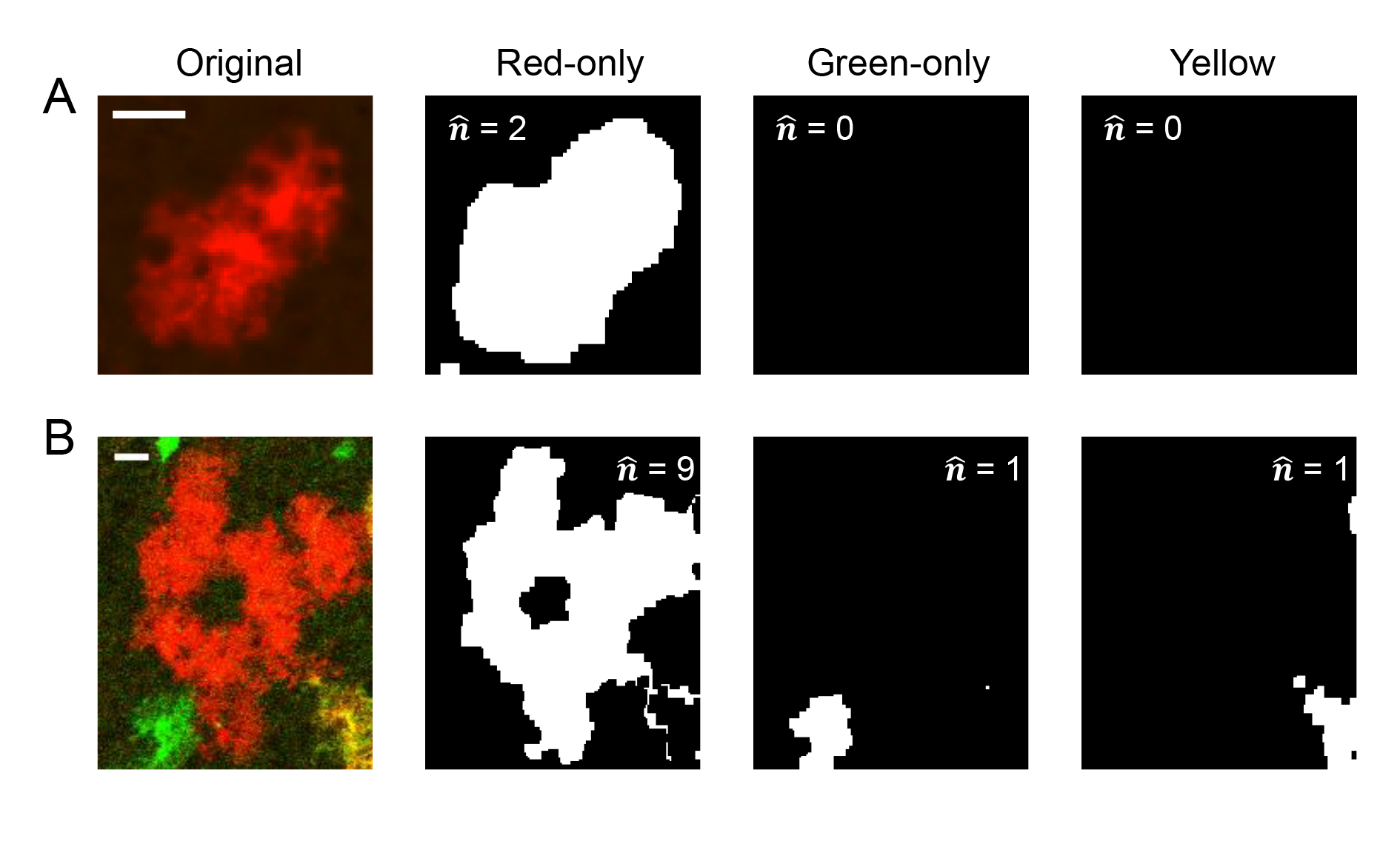

Supplement: S3 Fig — (A, B) Counting results of glia clusters from images acquired using a slide scanner and a CFM, respectively. Binary masks of cells regions were generated for each color by thresholding and morphological operations. Estimated cell numbers of each color are marked in the images. The glia cluster in A contains 2 red glia and the glia cluster in B contains 9 red glia, 2 green glia and 1 yellow glial cell according to ground truth annotations. Scale bars, 25 μm. (TIF) [file pone.0257426.s003.tif]
